# Effortless awareness: using real time neurofeedback to investigate correlates of posterior cingulate cortex activity in meditators' self-report

**DOI:** 10.3389/fnhum.2013.00440

**Published:** 2013-08-06

**Authors:** Kathleen A. Garrison, Juan F. Santoyo, Jake H. Davis, Thomas A. Thornhill, Catherine E. Kerr, Judson A. Brewer

**Affiliations:** ^1^Yale Therapeutic Neuroscience Clinic, Department of Psychiatry, Yale University School of MedicineNew Haven, CT, USA; ^2^Contemplative Studies Initiative, Clinical and Affective Neuroscience Laboratory, Department of Neuroscience, Brown UniversityProvidence, RI, USA; ^3^Department of Philosophy and Cognitive Science, City University of New York Graduate CenterNew York, NY, USA; ^4^Department of Family Medicine, Brown UniversityProvidence, RI, USA

**Keywords:** neurophenomenology, grounded theory, real time fMRI, meditation, posterior cingulate cortex, self-report, introspection, self-referential processing

## Abstract

Neurophenomenological studies seek to utilize first-person self-report to elucidate cognitive processes related to physiological data. Grounded theory offers an approach to the qualitative analysis of self-report, whereby theoretical constructs are derived from empirical data. Here we used grounded theory methodology (GTM) to assess how the first-person experience of meditation relates to neural activity in a core region of the default mode network—the posterior cingulate cortex (PCC). We analyzed first-person data consisting of meditators' accounts of their subjective experience during runs of a real time fMRI neurofeedback study of meditation, and third-person data consisting of corresponding feedback graphs of PCC activity during the same runs. We found that for meditators, the subjective experiences of “undistracted awareness” such as “concentration” and “observing sensory experience,” and “effortless doing” such as “observing sensory experience,” “not efforting,” and “contentment,” correspond with PCC deactivation. Further, the subjective experiences of “distracted awareness” such as “distraction” and “interpreting,” and “controlling” such as “efforting” and “discontentment,” correspond with PCC activation. Moreover, we derived several novel hypotheses about how specific qualities of cognitive processes during meditation relate to PCC activity, such as the difference between meditation and “trying to meditate.” These findings offer novel insights into the relationship between meditation and mind wandering or self-related thinking and neural activity in the default mode network, driven by first-person reports.

## Introduction

First-person subjective experience is critical for furthering our understanding of cognitive processes. Recent interest surrounds neurophenomenology—an approach that utilizes introspective self-report to inform the analysis and interpretation of objective physiological data related to consciousness and cognition (Varela, 1996; Lutz and Thompson, 2003). For functional neuroimaging studies, first-person reports of experience can be used to reduce the opacity of both the neural response and cognitive task strategy.

We recently conducted a real time functional MRI (rtfMRI) study of meditation to closely link the subjective experience of meditation with neuroimaging data in real time (Garrison et al., 2013). Adept meditators reported a significant correspondence between their moment-to-moment experience of meditation and real time neurofeedback from the posterior cingulate cortex (PCC), a brain region previously found to be activated during self-related thinking (Buckner et al., 2008) and deactivated during meditation (Brewer et al., 2011). Moreover, they were able to use what they had learned about the subjective qualities of meditation that related to feedback in order to volitionally deactivate the PCC. However, because the PCC has been associated with numerous cognitive states (Andrews-Hanna et al., 2010) the specific aspects of subjective experience that relate to PCC activity are yet unknown.

Here we use grounded theory methodology (GTM; Glaser and Strauss, 1967) to induce theory grounded in first-person data, consisting of meditators accounts of their experience during runs of the rtfMRI study, and third-person data, consisting of corresponding feedback graphs representing PCC activity during the same runs. GTM is a method of qualitative inquiry that seeks to generate theory from empirical data. Developed for use in sociology, GTM is now widely used across disciplines (e.g., Kennedy and Lingard, 2006), including the analysis of meditation diaries in clinical trials of Mindfulness Based Stress Reduction (e.g., Kerr et al., 2011). Here we use GTM to describe and quantify phenomenal subjective experience related to meditation. Specifically, the purpose of the current study was to investigate the subjective experience of meditation corresponding to PCC activity in adept meditators, in order to derive testable hypotheses for further inquiry.

## Materials and methods

### Participants

Ten experienced meditators participated in the study (7 male, 3 female; 9 right-handed, 1 ambidextrous; 9 White, non-Hispanic, 1 Hispanic; mean age 49.2 ± 12.5 years; mean education 19.2 ± 3.0 years). Meditators were experienced in different contemplative traditions including Theravada (*N* = 4), Zen (*N* = 3), Catholic Contemplative (*N* = 1), Catholic Contemplative and Zen (*N* = 1), and Gelugpa of Tibetan Buddhism (*N* = 1); and on average reported a total of 10,567 ± 4276 practice hours over 18.4 ± 4.9 years, comprised of daily practice and retreats. All participants provided informed consent for the study in accordance with institutional guidelines.

### Real time fMRI acquisition

We used a Siemens 1.5 Tesla Sonata MRI with standard eight-channel head coil to acquire a high-resolution anatomical scan, collected using a magnetization prepared rapid gradient echo (MPRAGE) sequence (*TR*/TE = 2530/3.34 ms, 160 contiguous sagittal slices, slice thickness 1.2 mm, matrix size 192 × 192, flip angle = 8°), and used to register data to the Montreal Neurological Institute (MNI) template brain (Mazziotta et al., 1995), which was used to define the overall reference coordinate system. Next a lower resolution T1-weighted anatomical scan was acquired (*TR*/TE = 500/11 ms, field of view = 220 mm, slice thickness = 4 mm, gap = 1 mm, 25 AC-PC aligned axial-oblique slices). An initial functional reference scan was acquired to register the PCC region of interest from MNI space. The PCC region of interest was defined based on peak deactivation in our previous study of meditation (MNI coordinates: −6, −60, 18) (Brewer et al., 2011). Functional images for feedback runs were then acquired beginning in the same slice location as the T1-weighted data, using a T2^*^-weighted gradient-recalled single shot echo-planar pulse sequence (*TR*/*TE* = 2000/35 ms, flip angle = 90°, bandwidth = 1446 Hz/pixel, matrix size = 64 × 64, field of view = 220 mm, voxel size = 3.5 mm, interleaved, 46 volumes), with the last volume discarded.

### Real time fMRI display

We used E-prime 1.2 (pstnet.com) to display a feedback graph representing the percent blood oxygenation level-dependent (BOLD) signal change in the PCC (corrected for global brain signal) during meditation relative to baseline (see Figures 4, 5 for examples). Real time image processing and feedback display for this study have been previously reported (Garrison et al., 2013). We note that image processing, from acquisition to feedback display, required less than 1 s.

### Real time fMRI protocol

Our rtfMRI protocol was designed to allow meditators to “discover” how a feedback graph representing activity in the PCC corresponded with their own subjective experience of meditation in real time. This protocol was comprised of a 4-step series of runs progressing from: (1) meditation with offline feedback (feedback graph shown offline after each run); (2) meditation on a graph with offline feedback; (3) meditation with real time feedback from the PCC; to (4) volitional manipulation of the feedback graph. This protocol was designed to progress from the most naturalistic setting for meditation (step 1, 4 runs), to meditation using a dynamic graph as the object of focus (step 2, 3 runs), to meditation with a graph of feedback from one's own brain in real time (step 3, 3 runs), to volitional manipulation of the feedback graph (step 4, 6 runs). Each run began with a 30 s baseline task, during which participants viewed adjectives and were asked to “think about and decide” if the words described them (Kelley et al., 2002). Similar tasks requiring evaluation of trait adjectives have been shown previously to engage self-related processing and regions of the default mode network including the PCC (Northoff et al., 2006). Here the active baseline task was used to provide a more stable baseline between groups, as we have previously found differences in PCC activity between meditators and non-meditators at rest (Brewer et al., 2011), and to provide a more stable baseline across runs within-subjects. Baseline was followed by a 1-min meditation task, with specific additional instructions per step, as described below.

### Real time fMRI instructions

#### Meditation with offline feedback

For the first meditation task, after the word task, the screen will go blank. This will be your cue to meditate for about 60 s. During the meditation, please pay attention to the physical sensation of the breath wherever you feel it most strongly in the body. Follow the natural and spontaneous movement of the breath, not trying to change it in any way. Just pay attention to it. If you find that your attention has wandered to something else, gently but firmly bring it back to the physical sensation of the breath. Please keep your eyes open.

#### Meditation on a graph with offline feedback

For the second meditation task, after the word task, you will see a graph start to form, that will fill in a new line every 2 s. This is an arbitrary graph, and does not show your brain activity. We ask that when you see the graph start to form, you again meditate for 60 s, here using the graph as your object of meditation—just paying attention to the graph as you would any other object of focus or concentration such as your breath. Pay attention to the graph, not trying to change it in any way. If you find that your attention has wandered to something else, gently but firmly bring it back to the graph. Please keep your eyes open.

#### Meditation with real time feedback

For the third meditation task, after the word task, you will see a similar graph start to form, and again we ask that you meditate for 60 s, using the graph as your object of meditation. Now the graph you see during the run will show relative activity in a particular region of your brain. Thus, for these runs, the graph you see during the run may correspond with your experience. There is a 2–4 s delay between your brain activity and the graph, thus if the graph does correspond with your experience, it will do so with a delay of 2–4 s. It may be helpful to look back at short stretches of time to notice your experience in relation to how the graph changes. We ask that you meditate, using the graph as your object of meditation, and now also notice your moment-to-moment experience in relation to how the graph changes.

#### Volitional manipulation of the feedback graph

Participants were first asked to volitionally decrease the feedback graph for 3 runs, using the following instructions: For the final task, after the word task, you will see a similar graph start to form that will show relative activity in a particular region of your brain, and may correspond with your experience. For these runs, we will ask you to use your mind to make the graph go blue. You may draw from your experience over the previous runs. You will have 60 s.

Participants were then asked to volitionally increase the feedback graph, using the following instructions: Finally, for 3 runs we will ask you to use your mind to make the graph go red. You may draw from your prior experience, and you'll have 60 s.

### Self-report

For steps 1, 2, after each run, meditators were first asked: (1) Please briefly describe your experience during the meditation. They were then shown a graph of their brain activity during the meditation (offline feedback) and asked: (2) On a scale from 0–10, how well does the graph correspond with your experience during the meditation, 0 being not at all, 10 being perfectly? and (3) How did you know? For step 3, after each run, meditators were asked the same questions, however, for these runs, they were asked to rate how well the graph they saw *during the run* (real time feedback) corresponded with their experience. For step 4, after each run, meditators were asked the same questions, and also to report: (4) What strategy did you use to make the graph go blue/red? Self-reports were audio recorded for offline transcription. Self-report for questions 1–4 were included in the current analysis.

Meditators practiced self-report for each step prior to actual scanning. Overall, they were instructed: In all of the meditation tasks, we are interested in your own experience of meditation, paying attention to an object of focus or concentration. After each run, we'll ask you to describe your own experience during the meditation period. In this study, we're interested in how activity in particular brain regions lines up with your experience of meditation. For (1) Please briefly describe your experience during the meditation, they were instructed: This question is open ended, but it's important to be concise, giving us the highlights of your experience during the meditation. For example, we may ask, “Was there anything different in your experience between the beginning, middle, and end of the meditation?” For (2) On a scale from 0–10, how well does the graph correspond with your experience during the meditation, they were instructed: The graph shows relative activity in a particular region of your brain over the meditation period. This graph may correspond with your moment-to-moment experience during the meditation. To make it easy to follow, values above the line will be red, and values below the line will be blue. We will ask you to look at the graph, and to consider how the graph does or does not correspond with your experience during the meditation. For example, we'll ask you to consider how the graph corresponds with your general experience of meditation, including mental effort, concentration, or mental state. Don't worry about every little detail, instead focusing on the more general aspects of these. For example, if you remember something about your experience at the beginning, middle or end of the meditation, you may look to see if your experience is reflected in how the graph changes. Any time you are shown a graph of your brain activity, the graph will show relative activity in a single brain region. We are only using one brain region for this study. For (3) How did you know, they were instructed: In other words, what about the graph does or does not correspond with your experience? Was there anything different in your experience that you notice corresponds to how the graph changes? We may refer to a specific aspect of the graph and ask “Did anything in particular correspond to this point on the graph?”

Meditators were asked five additional Likert item questions after each run. These ratings data are reported elsewhere (Garrison et al., 2013), but the questions are described here because they have the potential to influence meditators' self-reports. Questions included: (1) On a scale of 0–10, how distracted or focused were you during the meditation (0 = very distracted, 10 = very focused)? (2) On a scale of 0–10, how aware were you during the meditation (0 = not aware, 10 = very aware)? For this question, they were instructed: How aware were you of whatever arose in your moment to moment awareness? For example, you can be very aware that you are distracted. (3) On a scale of 0–10, how vivid was your experience (0 = not vivid, dull, hazy, fuzzy, 10 = vivid, sharp, clear, crisp)? (4) On a scale of −10 to 10, how was your mental state (−10 = sluggish or drowsy, 0 = relaxed but balanced, steady, even, alert, and 10 = agitated, racing, excited or restless)? (5) On a scale of 0–10, how was your mental effort (0 = effortless 10 = forced, pushed, tight, contracted)?

### Data analysis

Self-reports were transcribed verbatim by research assistants. The data consisted of these transcripts for each run and corresponding figures of feedback graphs of PCC activity from the same run. Qualitative data analysis was conducted following the principles of grounded theory (Glaser and Strauss, 1967), which entails an iterative process of data coding and analysis, outlined below. The goal of data analysis was to evaluate how self-reported experience corresponds with PCC activation or deactivation during meditation in experienced meditators.

**Figure 1:**
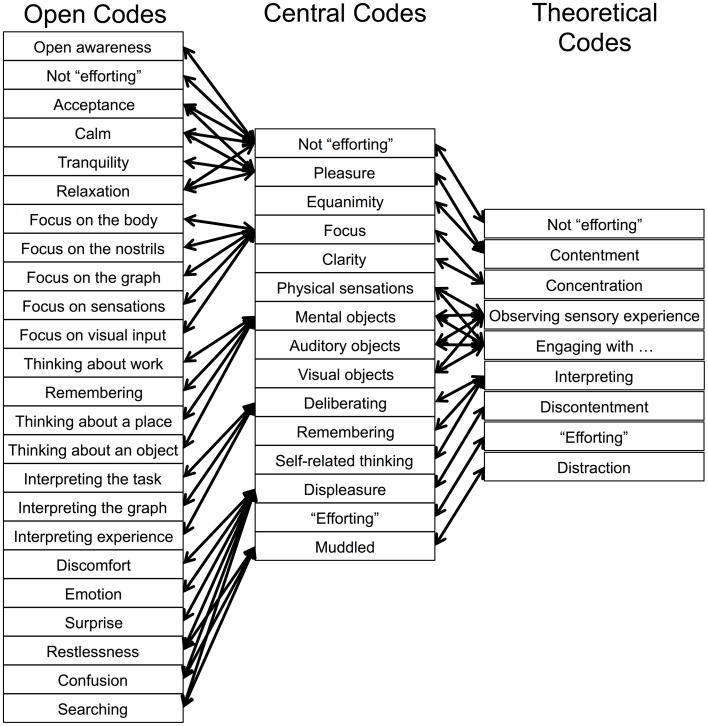
**Representation of the open codes, central codes, and theoretical codes derived from self-report and neurofeedback graph data using grounded theory methodology**.

*Initial or open coding* is used to identify and label words or phrases in the data (Birks and Mills, 2011). In our study, at the level of initial coding, data were analyzed as sets of self-report transcripts and graphs of PCC activity from the same run, in order to generate specific hypotheses about the relation between subjective experience and PCC activity. Initial coding involved reading each line of text while referring to the graph of PCC activity, and recording all instances of reference to the graph, either explicit (e.g., “the graph was blue”) or implicit (e.g., across time, “near the end of the run”), in order to capture meaning between the datasets at those instances. Both the first-person self-report data and the third-person brain imaging data were required to indicate PCC activation or deactivation for the initial code to be categorized as such. Excerpts were taken from the text, coded into a database, and labeled according to whether they corresponded with PCC activation or deactivation, as well as their specific content of meaning. For example, the excerpt “I noticed my breath and then my body somewhat, and then it started to go more blue” was labeled as “PCC deactivation,” “noticing the breath,” and “noticing the body.” In this way, initial coding was used to generate ideas by open coding many instances in the dataset (Figure 1).

*Focused coding* is used to group codes into conceptual categories as higher order codes (Birks and Mills, 2011). Focused coding employs a constant comparative method, whereby data are compared with emerging codes to identify patterns, revise terminology, combine, add, or eliminate codes, based on their prevalence, conceived importance, and relevance. For example, “noticing the breath” and “noticing the body” were combined into the open code “physical sensations” and sorted under the central code “focus.”

*Theoretical sampling* refers to resampling the data to return codes that only fit in existing categories (Birks and Mills, 2011). Datasets were resampled to ensure that emergent codes fit with the content of meaning of all of the relevant text excerpts under those codes. The aim is to ensure that the final theoretical codes are saturated in the data, so that any further analysis results in no new ideas or codes. At this stage we also recorded the frequency with which a given theoretical code was grounded in the data, i.e., the number of instances of self-report excerpts used to derive the coding structure leading to that theoretical code. Finally, we grouped theoretical codes into principal constructs that corresponded with PCC activation or deactivation.

All runs of the step-wise rtfMRI protocol were included in the analysis. However, the protocol was designed such that across steps, meditators could “discover” how their own experience of meditation corresponded to the feedback graph. This process involved getting used to meditating in the fMRI scanner (step 1), meditating while viewing a mock feedback graph (step 2), meditating while seeing a feedback graph from their own brain (step 3), and finally, volitionally manipulating the feedback graph (step 4). As expected, this learning process was represented in the self-report data, such that meditators reported more instances of getting used to the experimental paradigm in earlier runs (e.g., *“It took a moment to adjust to the sound and the looking, but as it progressed I felt more comfortable doing it”*). As such, though all runs of the stepwise protocol were included in open coding, the final stages of theoretical sampling focused on latter steps (steps 3, 4).

GTM was conducted by the second author (Juan F. Santoyo), a 21-year old Hispanic male undergraduate Neuroscience and Contemplative Science student, with no other role in the study. Prior to GTM, Juan F. Santoyo disclosed limited familiarity with literature related to the PCC (including Brewer et al., 2011) but no preconceived notion of PCC function; and a personal meditation practice (including Mahāyāna, Theravada, Mahasi, classical Daoist, and mindfulness), which provided him a bias through which to directly interpret the self-report data on introspection (as suggested by Wallace, 2000). As part of GTM, he composed memos of the coding process and emergent ideas, to both stimulate and provide a record of the coding process, and to allow for regular second-person cross-checking of emergent ideas with co-authors. This allowed for independent evaluation of the qualitative analysis. GTM is generally carried out by an individual researcher, with explicit acknowledgment of the role of the individual in generating hypotheses from the data (Mills et al., 2006). Hypotheses are derived from the data using an iterative process of coding and memo writing, with coding strategies, the emergent coding framework, and interpretation of data cross-checked by co-authors. In this way, elements of multiple coding provide cross-checking of grounded theory without multiple coding of the dataset (Barbour, 2001).

## Results

**Figure 2:**
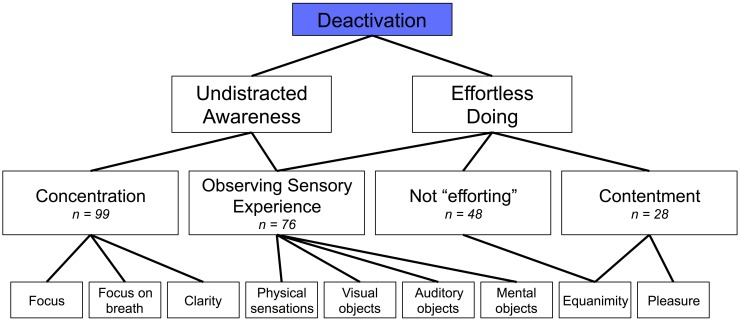
**Phenomena of the subjective experience of meditation related to posterior cingulate cortex deactivation (*n* = number of occurrences in self-reports)**.

**Figure 3:**
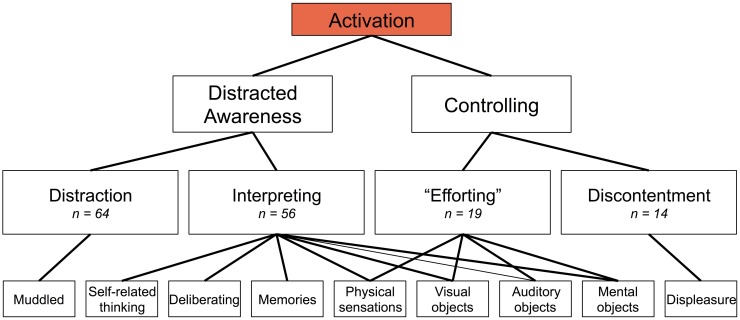
**Phenomena aspects of the subjective experience of meditation related to posterior cingulate cortex activation (*n* = number of occurrences in self-reports)**.

**Figure 4:**
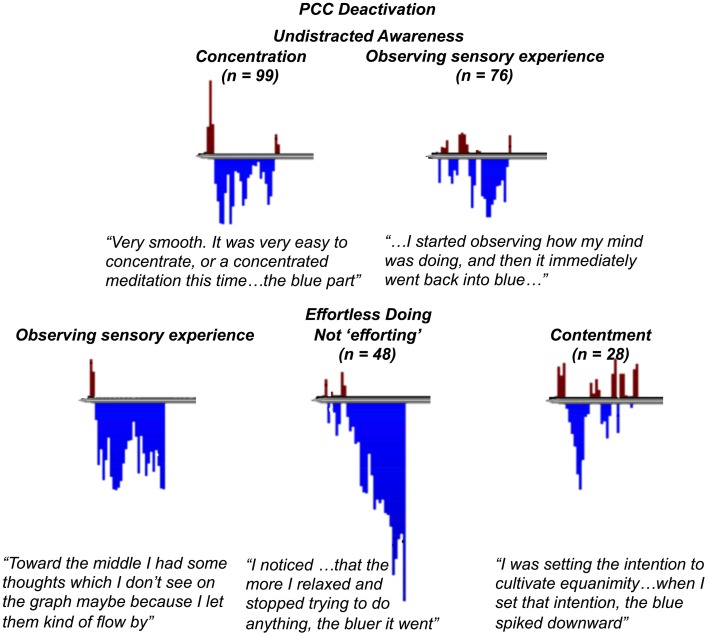
**“Undistracted awareness” and “effortless doing” as basic eliciting factors of posterior cingulate cortex deactivation.** Examples of self-report transcripts and feedback graphs for the theoretical codes leading to the basic eliciting factors of “undistracted awareness” **(top)** and “effortless doing” **(bottom)**.

**Figure 5:**
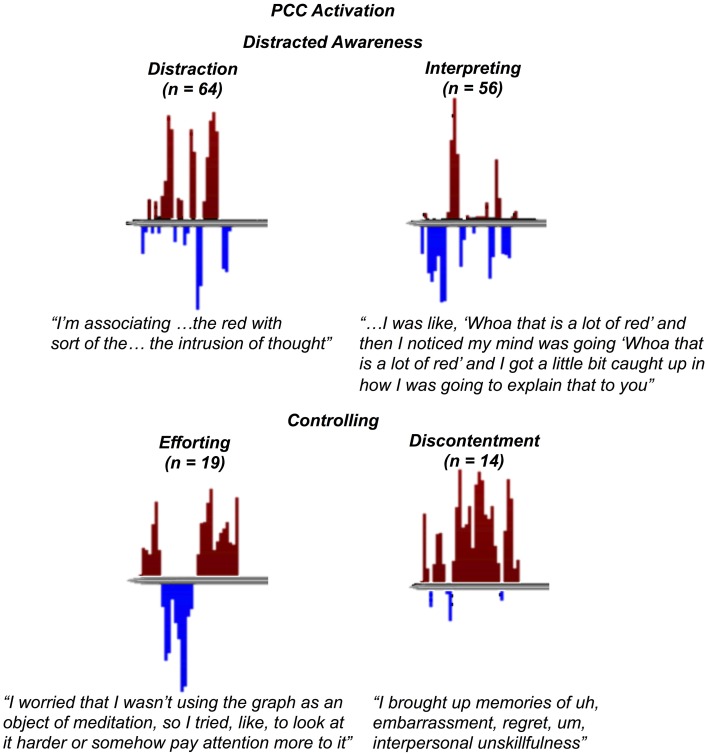
**“Distracted awareness” and “controlling” as basic eliciting factors of posterior cingulate cortex activation.** Examples of self-report transcripts and feedback graphs for the theoretical codes leading to the basic eliciting factors of “distracted awareness” **(top)** and “controlling” **(bottom)**.

The open codes, central codes, and theoretical codes derived from the data using GTM are displayed in Figure 1. From these, we determined principal constructs for the phenomena of subjective experience that corresponded with PCC deactivation (Figure 2) or PCC activation (Figure 3). Specific examples of data grounding the principal constructs are provided in Figures 4, 5.

### “Undistracted awareness” and “effortless doing” as basic eliciting factors of pcc deactivation

Meditators reported phenomena in their subjective experience related to “undistracted awareness” or “effortless doing” as basic eliciting factors of PCC deactivation (Figure 2).

“Undistracted awareness” emerged from data related to settled, concentrated, or clear attention to momentary experience, and is comprised of the theoretical codes for “concentration” and “observing sensory experience” (Figure 4). For “concentration” meditators reported instances of single-pointed concentration such as focus on the breath (i.e., concentration on the task) or open focus marked by a quality of clarity (*n* = 99 instances). For “observing sensory experience” in the context of “undistracted awareness,” meditators reported instances of noticing sensory stimulus–such as visual stimulus, physical sensations, or thoughts—but not being distracted by their sensory experience (*n* = 76).

“Effortless doing” emerged from data related to a calm, tranquil, relaxed, and effortless way of doing things, and is comprised of the theoretical codes for “observing sensory experience,” “not efforting,” and “contentment” (Figure 4). For “observing sensory experience” in the context of “effortless doing,” meditators reported instances of paying attention to sensory stimulus but not engaging with their sensory experience by deliberative thinking or action (*n* = 76 total). For “not efforting” meditators reported instances of relaxation without effort and without any attempt to control their experience, such as when they would just “let go” and meditate without trying to make anything happen (*n* = 48). For “contentment,” meditators reported instances of satisfaction or acceptance of things as they are, feelings of ease, equanimity, or bliss (*n* = 28).

### “Distracted awareness” and “controlling” as basic eliciting factors of PCC activation

Meditators reported phenomena in their subjective experience related to “distracted awareness” or “controlling” as basic eliciting factors of PCC activation (Figure 3).

“Distracted awareness” emerged from data related to distraction, lack of concentration, or unsettled awareness, such as the awareness that one's mind is wandering or thinking and that one is unable to control these processes (i.e., carried away in thoughts or experience), and is comprised of the theoretical codes for “distraction” and “interpreting” (Figure 5). For “distraction,” meditators reported instances of distraction or lack of focus, such as when they were unable to pay attention during the run or when they reported feeling hazy, unclear, or muddled (*n* = 64). For “interpreting,” meditators reported instances of thinking, deliberating, or remembering, such as trying to understand the graph or rehearsing self-report (*n* = 56).

“Controlling” emerged from data related to trying to change the way things are or affect experience, often associated with a dissatisfaction with current experience, and is comprised of the theoretical codes for “efforting” and “discontentment” (Figure 5). For “efforting,” meditators reported instances of exerting effort in order to make something happen or change one's experience, such as *trying* to pay attention or trying to change the graph (*n* = 19). For “discontentment,” meditators reported instances of feeling unhappy, uncomfortable, or in some way dissatisfied or displeased, such as feeling unpleasant emotions such as anger, wanting the experiment to end, or feeling frustrated with the feedback graph (*n* = 14).

## Discussion

In this study, we used GTM to analyze self-reports of experience and graphs of real time neurofeedback in order to evaluate the phenomena of subjective experience that corresponds with PCC activity for experienced meditators. We used GTM to derive testable hypotheses about the relationship between subjective experience and PCC activity that are grounded in the data. We found that for meditators, the principle constructs of “undistracted awareness” and “effortless doing” corresponded with PCC deactivation, whereas “distracted awareness” and “controlling” corresponded with PCC activation.

These findings are consistent with prior work indicating that the PCC is activated during mind wandering (Mason et al., 2007) and self-referential processing (Whitfield-Gabrieli et al., 2011) such as past and future thinking (Andrews-Hanna et al., 2010), and deactivated during three meditation practices (concentration, loving kindness, and choiceless awareness) in expert compared to novice meditators (Brewer et al., 2011; Pagnoni, 2012). Our primary constructs for the basic eliciting factors of PCC activity fit well with these prior findings of PCC activation related to mind wandering and PCC deactivation related to meditation. From the data, subjective reports of “distracted awareness” corresponding with PCC activation included instances of thinking about the past or future (e.g., “*I began by thinking about a variety of things that need to be done, emails that need to be sent, things that I have not done in a timely fashion, that type of thing*”) and mind wandering (e.g., *“I got caught up in thinking what I was going to tell you”*). Likewise, subjective reports of “undistracted awareness” corresponding with PCC deactivation included instances of concentration meditation (e.g., “*I felt much more focused on my breath and felt like I had fewer moments of distraction or interruption”*).

Beyond confirming previous studies, we demonstrate that rtfMRI with self-report can be used to generate new data-driven and testable neurophenomenological hypotheses about particular brain regions; in this case the PCC. rtfMRI neurofeedback improves the temporal resolution and specificity between subjective experience and brain activity, as many cognitive processes may be present at any one moment. The GTM approach is distinct from other neurophenomenology studies in which for example subjects are provided with intensive training on how to self-report (e.g., Lutz et al., 2002). Below we discuss three emergent hypotheses that best exemplify the strengths of the method, the potential implications, and the need for further neurophenomenological investigation.

One hypothesis emerged regarding specific qualities of self-related processing related to PCC activation. Of particular interest in regard to previous associations with mind-wandering (e.g., Mason et al., 2007; Whitfield-Gabrieli et al., 2011), several meditators reported instances of mind wandering that *did not* elicit PCC activation, or, likewise, reported using a strategy of mind wandering or self-related thinking in unsuccessful attempts to activate the PCC:

Meditator 134 (run 12): For this meditation, now I just tried not to push it at all, I just wanted to see what would happen with just really resting, not visualizing anything, not using anything as a tool, just opening up the space and resting, and I think towards the middle I had some thoughts which I don't see on this graph maybe because I just let them kind of flow by, but I noticed some thoughts. But in general, it just felt a little bit more restful than the last [run].

Meditator 141 (run 14): I was surprised that [the graph] was so blue on that second part. I was observing a lot of what I was thinking, but I was thinking about a lot of things, for example, what I had to do the rest of the day.

These and similar instances of mind wandering that *did not* lead to PCC activation suggest that the PCC may be involved in more subtle aspects of experience related to thinking rather than just the thoughts themselves (e.g., Andrews-Hanna et al., 2010). Mind wandering or self-related thinking that *does not* lead to PCC activation may be distinguished by a quality of not being pushed, pulled, or lost in mental content, feelings, or thoughts as they arise, described by meditators as “letting things flow by” or “observing thinking.” In contrast, mind wandering or self-related thinking *leading to* PCC activation may have a quality of reactivity to mental content or thoughts, such as desire or aversion toward mental content, ruminative thinking, or “getting caught up in narrative.” As the PCC becomes activated during craving and emotion (Garavan et al., 2000; Kober et al., 2008), further studies may also test whether the particular qualities of self-related thinking leading to PCC activation influence how self-referential processing and mind wandering lead to stress and disease (e.g., Killingsworth and Gilbert, 2010).

Another hypothesis emerged regarding specific qualities of meditation practice related to PCC deactivation. Here, several meditators made an explicit distinction between “effortful” attempts to meditate associated with PCC activation, and “effortless” meditation associated with PCC deactivation:

Meditator 140 (run 11): The biggest thing I noticed was that the more I relaxed and the less I did, the bluer [the graph] went … the more I relaxed and stopped trying to do anything, the bluer [the graph] went.

Meditator 123 (run 9): The red bars correspond to times when I was trying to either force the experience or trying to think about, thinking about stuff in general, thinking about making [the graph] blue. And then when I could let it go, [the graph] turned blue.

These and similar instances distinguish between “effortful” and “effortless” meditation, represented in the principle constructs of “controlling” leading to PCC activation, and “effortless doing” leading to PCC deactivation. These data suggest that “trying to meditate” may be associated with PCC activation, whereas “not trying” or effortless meditation may be associated with PCC deactivation. Such a distinction may be instructive for meditation training.

Recently, Pearson et al. proposed that the PCC is involved in signaling environmental change and shifts in behavior (Pearson et al., 2011), whereby decreased PCC activity reflects operation within a current cognitive set, and increased activity reflects a change in environment (external or internal) and “promotes flexibility, exploration, and renewed learning.” Our data provide some support for this, as we found consistent PCC deactivation associated with concentration, staying within the framework of a current cognitive set. The change in experience related to concentration may be when meditators force their concentration, associated with PCC activation. If one were to interpret “efforting” as related to inflexibility, rather than flexibility and learning, our data do not provide support for Pearson's assertion that increased PCC activity promotes flexibility. Future studies using rtfMRI may directly test this.

Another distinction of interest emerged regarding meditators reports of “sensory experience,” which were associated with *both* PCC activation and deactivation. Through the constant comparative method of GTM, we found that PCC activation was associated with reports of being distracted by, reacting to, or trying to control sensory objects (physical, visual, auditory, or mental objects such as thoughts):

Meditator 141 (run 7): Especially when [the graph] started getting really really red and I was like “Whoa that is a lot of red” and then I noticed my mind was going “Whoa that is a lot of red.”

Meditator 138 (run 14): I tried to bring my perception away from the breath and more towards the visual and that brought [the graph] back into the red at the end.

In contrast, PCC deactivation was associated with reports of concentration on, or awareness or observation of sensory experience:

Meditator 134 (run 13): Toward the middle I began to experience a tingling through my body and so I was just kind of watching that for a while.

Meditator 62 (run 10): I maintained primary awareness on the full range of experience, including, just, awareness of the body and various touch points, the breath moving throughout the body, the sound being integrated into that sort of, sort of fuller awareness while watching the colors with relative ease … body awareness.

These data suggest that sensory experience related to “distraction” or “controlling” is associated with PCC activation, whereas sensory experience related to “undistracted awareness” or “effortless doing” is associated with PCC deactivation. This distinction was in part task-related, as meditators were asked to use dynamic sensory experience—both their breath and the feedback graph—as the object of meditation. Meditators had to learn to be on task—to pay attention to the breath and the graph—while meditating, i.e., not being distracted by, interpreting, or controlling the breath or the graph.

The current study drew meditators from a variety of contemplative traditions including Catholic contemplative, Theravada, Zen, and Tibetan Buddhism. Despite this variation, consistent hypotheses were derived that are in agreement with various traditional characterizations of the meditative state. For example, at an advanced stage of practice, as one Theravada Buddhist teacher described, “There will arise knowledge perceiving evident bodily and mental processes in continuous succession quite naturally, as if borne onward of itself … in the act of noticing, effort is no longer required to keep formations before the mind or to understand them” (Sayadaw, 1994). This consistency may extend to instructions for Mahamudra training in Tibetan Buddhism which include, “Do not pursue the past. Do not usher in the future. Rest evenly with present awareness,” and “Not meditating. Not analyzing. Just place the mind in its natural state” (Karma Wangchug Dorje, in Dunne, 2011). Thus, the construct of effortless awareness seems to be evident in both traditional descriptions of meditation practice, and in subjective reports of meditation related to neural activity in adept meditators.

To further refine these hypotheses, additional neurophenomenological studies can be conducted, driven by first-person reports, whereby meditators are asked to distinguish (over ongoing or successive real time feedback runs) between specific aspects of their experience that are closely related but differ in whether they elicit PCC activation or deactivation. Qualities of “self-related thinking” or “trying/not-trying to meditate” can be manipulated and reported upon by the individual, and further emergent categories tested and refined across subjects. Further studies will also investigate other regions of the default mode network, as well as large-scale brain systems.

## Limitations

An advantage of the approach was to include meditators, who are highly trained at first-person methods such as introspection (Fox et al., 2012) and who are able to gain access to different aspects of their experience (Lutz and Thompson, 2003). However, generalizability to novices or non-meditators may be limited given that meditators have a prior context—their contemplative tradition—within which to interpret both their meditation and the neurofeedback. Just as contemplative training may enable meditators to more carefully examine particular aspects of their experience, training may also bias them to evaluate only certain aspects of their experience. Moreover, meditators were presented with Likert items after each run (e.g., *how was your mental effort?*) that may have influenced introspection. Related to this, although we followed the standards of GTM, it is possible that our coder introduced interpretive bias, and a validation of the observed relationships with newly acquired data across different coders will improve reliability of our findings. Nevertheless, our findings offer testable hypotheses for further study in meditators and other groups such as novices, and clinical populations such as stress.

In this study, demand characteristics may result from asking meditators to look for correspondence between their experience and the feedback graph. To minimize these, participants were always asked to consider how the graph *did or did not* correspond with their experience, and it was emphasized in training that we were interested in how *their own experience of meditation* corresponded with the graph. Related to this, first-person self-reports may have been influenced by the feedback graph. During steps 1, 2 in which meditators were provided offline feedback after they had already described their experience, this potential influence was overt, for example, “*There's this one place where I was getting lost, and I wonder if I'm wrong about where it was [on the graph]? If it was closer to the beginning, then I can see where there might be a place just a little ways in where [the graph] goes back up? … I'm doubting myself where exactly that was.”* Our real time feedback protocol was developed in an attempt to minimize this potential confound (Garrison et al., 2013). Offline feedback was provided in order to enable meditators to “discover” how their own experience related to the feedback graph. For grounded theory, it was useful to have both first-person self-reports uncontaminated by third-person data (steps 1, 2), as well as self-reports enriched by evaluation related to real time neurofeedback (steps 3, 4). Real time feedback also allowed us to investigate the circular causality whereby (1) the ongoing first-person experience (meditation) modulates the third-person data (feedback graph), and (2) the content of the third-person data (feedback) affects the moment-to-moment first-person experience, and so on.

Finally, other work using electroencephalography (EEG) has shown that there is a fast on/off switch for task-related activation/deactivation of the PCC (Ossandon et al., 2011), suggesting that PCC activity may represent lower-level processing below that of conscious awareness, especially in individuals not trained to be aware of subtle aspects of experience. In the current study, the temporal resolution of rtfMRI did not allow us to similarly examine PCC activity around the task of meditation with this degree of temporal precision. Though efforting arose as its own category separate from distraction in our analysis, in some cases, PCC activity may be related to distraction, but perceived subjectively as efforting as meditators notice they are distracted and try to counteract distraction. Additionally, the PCC may be a marker of efforting but the actual effort to redirect attention or counteract distraction is likely subserved by other brain regions involved in cognitive control, for example the dorsolateral prefrontal cortex (Brewer et al., 2011; Allen et al., 2012). As the PCC was the only region that was analyzed here, future studies using EEG may be used to test these hypotheses.

## Conclusions

Using a neurophenomenological approach with grounded theory analysis, we described and quantified several aspects of the subjective experience of meditation related to PCC activity in adept meditators. “Undistracted awareness” and “effortless doing” were associated with PCC deactivation, whereas “distracted awareness” and “controlling” were associated with PCC activation. First-person reports of the subjective experience of meditation provided new insights into more refined aspects of meditation and self-related thinking associated with PCC activity, such as the difference between meditation and “trying to meditate.” These findings demonstrate the utility of our combined approach to generate hypotheses about cognition for further studies.

### Conflict of interest statement

The authors declare that the research was conducted in the absence of any commercial or financial relationships that could be construed as a potential conflict of interest.
